# Outcomes of patients in nasopharyngeal adenoid cystic carcinoma in the IMRT era: a single-center experience

**DOI:** 10.1186/s12885-024-12159-z

**Published:** 2024-05-10

**Authors:** Wen-Bin Wu, Wu-Lin Cai, Ye-Hao Zou, Rui You, You-Ping Liu, Zhao-Di Yuan, Qiong Li, Wen-Chao Li, Zhi-Xuan Pi, Yu-Long Xie, Kai Wen, Ming-Yuan Chen, Rui Sun

**Affiliations:** 1 Department of Nasopharyngeal Carcinoma, Sun Yat-Sen University Cancer Center, 651 Dongfeng East Road, Guangzhou, Guangdong 510060 P.R. China; 2 Sun Yat-Sen University Cancer Center; State Key Laboratory of Oncology in South China; Collaborative Innovation Center for Cancer Medicine; Guangdong Key Laboratory of Nasopharyngeal Carcinoma Diagnosis and Therapy, Guangzhou, 510060 China; 3 Department of Clinical Medicine, Zhongshan School of Medicine, Sun Yat-Sen University, Guangzhou, 510060 China

**Keywords:** Nasopharyngeal adenoid cystic carcinoma, Treatment modality, Surgery, Radiotherapy

## Abstract

**Objective:**

Nasopharyngeal adenoid cystic carcinoma (NACC) is a rare malignancy with special biological features. Controversies exist regarding the treatment approach and prognostic factors in the IMRT era. This study aimed to evaluate the long-term outcomes and management approaches in NACC.

**Methods:**

Fifty patients with NACC at our institution between 2010 and 2020 were reviewed. Sixteen patients received primary radiotherapy (RT), and 34 patients underwent primary surgery.

**Results:**

Between January 2010 and October 2020, a total of 50 patients with pathologically proven NACC were included in our analysis. The median follow-up time was 58.5 months (range: 6.0–151.0 months). The 5-year overall survival rate (OS) and progression-free survival rate (PFS) were 83.9% and 67.5%, respectively. The 5-year OS rates of patients whose primary treatment was surgery and RT were 90.0% and 67.3%, respectively (log-rank *P* = 0.028). The 5-year PFS rates of patients whose primary treatment was surgery or RT were 80.8% and 40.7%, respectively (log-rank *P* = 0.024). Multivariate analyses showed that nerve invasion and the pattern of primary treatment were independent factors associated with PFS.

**Conclusions:**

Due to the relative insensitivity to radiation**,** primary surgery seemed to provide a better chance of disease control and improved survival in NACC. Meanwhile, postoperative radiotherapy should be performed for advanced stage or residual tumours. Cranial nerve invasion and treatment pattern might be important factors affecting the prognosis of patients with NACC.

**Supplementary Information:**

The online version contains supplementary material available at 10.1186/s12885-024-12159-z.

## Introduction

Nasopharyngeal carcinoma (NPC) is a head and neck malignancy with high incidence in Southeast Asia, particularly in Southern China [1, 2]. The main histologic type of NPC is nonkeratinizing squamous cell carcinoma, and sequential chemoradiotherapy is the fundamental treatment. However, nasopharyngeal adenoid cystic carcinoma (NACC) rarely occurs in the nasopharyngeal cavity [3]. Clinically, management of NACC is rather difficult because of its insidious local growth pattern, insensitivity to radiotherapy, propensity for cranial nerve involvement, and relatively high incidence of distant metastasis [4–6].

Differing from nonkeratinizing squamous cell carcinoma in nasopharyngeal cancer, NACC is regarded as a high-grade neoplasm; consequently, the choice of treatment is radiotherapy (RT) alone, surgery alone, or surgery followed by RT [7, 8]. Previous studies have reported the treatment pattern and prognosis of NACC. However, the patient numbers enrolled in these studies are rather small because of the rare occurrence [9–15]. The two largest retrospective studies demonstrated conflicting results. One study with 26 patients demonstrated an overall survival (OS) benefit for patients receiving combined modality therapy (CMT) versus RT [10]. However, another study with 36 patients demonstrated no difference in OS between CMT and RT [9]. These retrospective studies were based on data over a long period including patients who underwent 2-dimensional radiotherapy (2DRT), and the inconsistency of treatment modality may influence the prognosis. With the common adoption of intensity-modulated radiation therapy (IMRT), the treatment patterns of NACC have changed. Modern IMRT systems are better able to target gross tumours while simultaneously protecting normal tissue compared with conventional 2DRT. The purpose of this study was to evaluate the treatment outcomes of patients with NACC after treatment with primary RT or primary surgery in the IMRT era.

## Materials and methods

The medical records of patients diagnosed with NACC at the Cancer Center of Sun Yat-sen University between January 2010 and October 2020 were retrospectively reviewed. All patients were considered valuable if they had data about patient demographics, pathological diagnoses, tumour details, treatment outcomes, and follow-up in our centre. Disease in all patients was reclassified according to the American Joint Committee on Cancer (AJCC) classification system (edition published in 2010) [16]. Diagnostic evaluation at presentation should include a complete physical examination, ultrasonography, chest X-rays, CT, PET-CT and MRI scans to evaluate the primary site and to exclude metastatic disease. Patients with primary NACC were included, whereas those with nasopharyngeal metastases from primary adenoid cystic carcinoma in other sites were excluded from this study. The plasma EBV DNA levels of patients were measured using quantitative polymerase chain reaction (qPCR) before the initiation of treatment [17].

The eligibility criteria for this study were as follows: (1) histologically confirmed adenoid cystic carcinoma (ACC), (2) no distant metastasis, (3) no previous treatment, (4) treatment administered with radical intent and (5) duration of follow-up longer than 6 months, (6) patients who had previously received primary treatment at an external institution, and for whom treatment details were unavailable, were not included in this study. The Ethics Committee at our Institute approved the study with a waiver of documented informed consent/assent.

### Treatment

#### Primary surgery

In the context of this study, “primary surgery” refers to the initial surgical intervention carried out at the time of diagnosis or presentation of the medical condition under investigation. In this study, 34 patients underwent primary surgery. The nasopharyngectomy (ENPG) procedure was performed for these patients. The resected nasopharyngeal tumours were removed via the nasal cavity, and the removed tissues and surgical margins were sent for pathological examination. If NACC patients had cervical lymph node metastases, selective neck dissection was also performed, followed by ENPG. Secondary surgery or postoperative radiotherapy (PORT) with or without chemotherapy would be encouraged and performed as a part of the whole treatment if multiple surgical margin biopsies were pathologically proven to be positive after the operation. Indications for PORT included a large primary tumour, nerve invasion, positive surgical margins, or the surgeons considered the primary tumour unresectable. In patients with large tumours invading deep tissues, nerves, the cavernous sinus or the skull base that could hardly be completely resected, gross tumour volume (GTV) included these sites.

#### Primary radiotherapy

In the context of this study, “primary radiotherapy” denotes the initial and primary utilization of radiotherapy as the standalone or primary modality of treatment. In this study, 16 patients received primary radiotherapy. Radiation was administered once per day for 5 days each week. The dose administered to the gross disease was 70 to 72 Gy at 2.0 to 2.3 Gy/fraction, as defined by clinical examination, head and neck CT or MRI, and, when indicated, PET-CT. Tissue volumes at risk of harbouring subclinical disease, including the bilateral neck, received 66 to 70 Gy at 2.0 to 2.3 Gy/fraction of IMRT. Chemotherapy and salvage surgery were also used as multidisciplinary treatments at the physician’s discretion.

#### Follow‑up

All patients were followed up to assess the disease status and performance status every 3 months in the first 3 years after treatment, every 6 months in the fourth and fifth years, and annually thereafter.

#### Statistical methods

Categorical variables were compared by the chi-square test or Fisher’s exact test. The time period between the start of treatment and death or progressive disease was used to calculate the OS and PFS, respectively. The Kaplan‒Meier method and the log-rank test were used to test for differences in the survival functions between strategies, as defined by clinical variables. To identify predictors of outcome, we performed a univariable analysis for each of the following variables: age, sex, alcohol history, tobacco history, nerve invasion, bone invasion, vessel invasion, lymph node metastasis, concurrent chemotherapy, and treatment pattern. We applied a process of several steps to develop a final model. The first step was to study the correlation between OS and PFS and each covariate via a univariable model followed by a preliminary multivariable Cox proportional hazards regression model. Thus, covariates with a univariable *P* < 0.1 were included in a preliminary multivariable Cox proportional hazards regression model. Variables that remained statistically significant (*P* < 0.05) were included in the final multivariable model.

All statistical testing was two tailed. Alpha was set at 0.05 for significance. All statistical testing was completed using SPSS software (Statistical Package for the Social Sciences version 25.0; Chicago, IL, USA) and the R language environment for statistical computing version 3.1.3 (open source).

## Results

Between January 2010 and October 2020, a total of 50 patients with pathologically proven NACC were included in our analysis, including 28 males and 22 females. The median age was 47 (range from 28 to 68. According to the AJCC 2010 criteria, 13 patients were grouped into stage I and stage II, and 37 patients were grouped into stage III and stage IV. Twenty-five (50%) patients had cervical lymph node metastasis (LNM) at diagnosis by physical and radiographical examination. Cranial nerve invasion existed in 42% (21/50) of all patients, and bone invasion existed in 72% (36/50) of all patients (Table 1).

**Table 1:** Characteristics and clinical data of the patients

Patient characteristics	Total n (%)	Surgery	RT	*P* Value
Total, N	50	34	16	
Age, y				0.697
≤ 47	23	15	8	
> 47	27	19	8	
Sex				0.838
Male	22	15	7	
Female	28	17	9	
Alcohol history				0.725
No	39	27	12	
Yes	11	7	4	
Tobacco history				0.562
No	37	26	11	
Yes	13	8	5	
Nerve invasion				0.432
No	36	21	8	
Yes	21	13	8	
Bone invasion				0.318
No	14	11	3	
Yes	36	23	13	
Vessel invasion				0.716
No	42	29	13	
Yes	8	5	3	
T stage				0.423
T1-T2	13	10	3	
T3-T4	37	24	13	
Lymph node metastasis				0.069
No	25	20	5	
Yes	25	14	11	
Concurrent chemotherapy				0.808
No	12	8	4	
Yes	38	26	12	

**Fig. 1:**
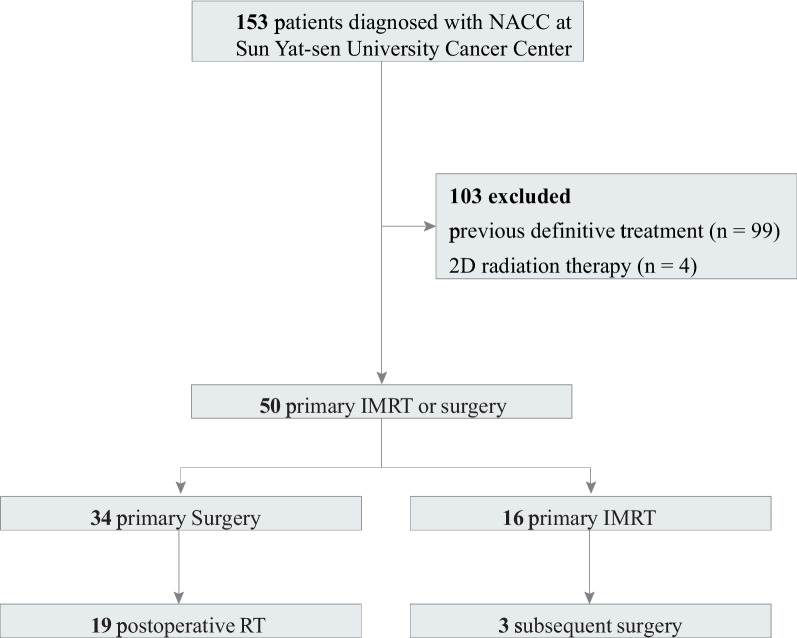
Treatment group schemes. Flowchart describing definitive treatment disposition. IMRT, intensity-modulated radiation therapy; CRT, chemoradiotherapy; RT, radiotherapy

Out of the 34 patients who underwent primary surgery, 19 subsequently received postoperative radiotherapy (RT). The median radiation dose administered was 62.24 (60–66) Gy (Fig. 1), 14 of them received chemotherapy, including 12 patients who received postoperative adjuvant chemotherapy and 2 patients who received preoperative induction chemotherapy. The percentage of patients with nerve invasion in the surgery group was 61.76%, compared to 73.33% in the surgery plus PORT group (*p* = 0.296). Bone invasion was observed in 32.35% of surgery patients and 67.65% of surgery plus PORT patients, with significant difference (*p* = 0.030). Comparisons regarding tumor staging and lymph node metastasis did not yield significant differences (T staging: *p* = 0.068; lymph node metastasis: *p* = 0.314) (Table S1).

**Fig. 2:**
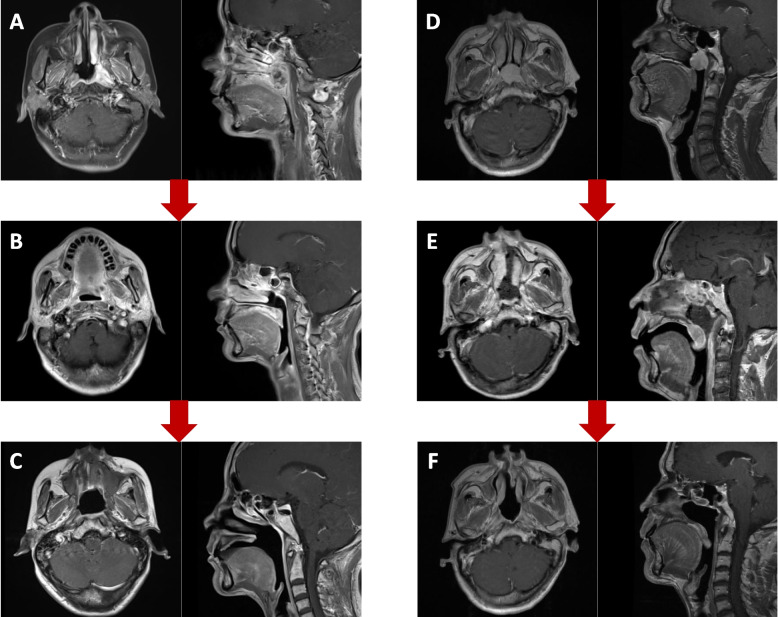
Response to therapy and survival outcomes. Gadolinium–enhanced magnetic resonance images taken (**A**) at the time of NACC before primary RT, (**B**) of residual tumour after primary RT, (**C**) at 6 months of subsequent surgery, (**D**) at the time of NACC before primary surgery, (**E**) at 1 week of primary surgery, and (**F**) at 6 months of postoperative RT. RT, radiotherapy; NACC, nasopharyngeal adenoid cystic carcinoma

Sixteen patients received primary radiotherapy (RT). The objective response rate (ORR) was 81.3% (13/16) after irradiation, including complete response (CR) in 4 cases and partial response in 9 cases. Three of them underwent subsequent surgery for the residual tumour after radiotherapy (Fig. 2), among which complete local resection with negative margins was achieved in 66.7% (2/3) of patients. All 16 patients were treated with IMRT at a daily dose range of 2.0–2.3 Gy for the primary tumour, and the prescription dose was 70–72 Gy. Twelve of them received chemotherapy, including 3 patients who received adjuvant chemotherapy and 9 patients who received induction chemotherapy.

**Fig. 3:**
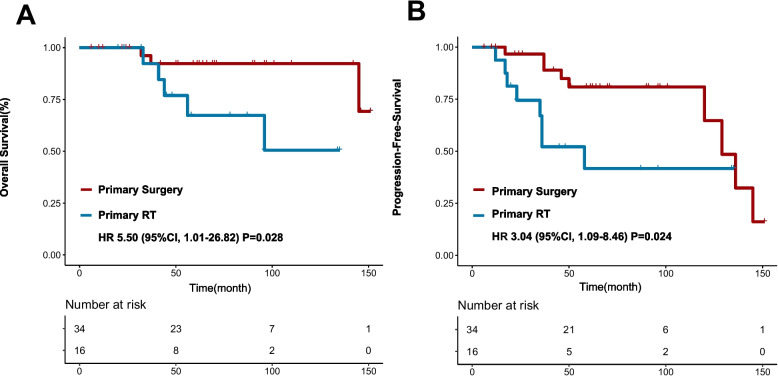
The overall survival rate (**A**) and progression-free survival rate (**B**) in patients with NACC between primary surgery and primary RT. RT, radiotherapy

The median follow-up time was 58.5 months (range: 6.0–151.0 months). The 5-year overall survival rate (OS) and progression-free survival rate (PFS) were 83.9% and 67.5%, respectively. On analysis of survival based on the different primary treatments, the 5-year OS of patients whose primary treatment was surgery or RT was 90.0% and 67.3%, respectively (log-rank *P* = 0.028), and the 5-year PFS of patients whose primary treatment was surgery or RT was 80.8% and 40.7%, respectively (log-rank *P* = 0.024). Compared to patients without nerve invasion, those with positive nerve invasion at diagnosis were more likely to develop progressive disease. The PFS of the two groups was 78.4% and 49.5% at 5 years (log-rank *P* = 0.011) (Fig. 3). To adjust the risk between different treatment groups, we introduced variables with prognostic potential, as indicated by univariable analyses, to a multivariable model. In patients who had nerve invasion, the adjusted HR was 2.903 (95% CI, 1.037–8.142; *P* = 0.034) for PFS. The adjusted hazard ratios of 5.497 (95% CI, 1.007–26.817; *P* = 0.049) for OS and 2.903 (95% CI, 1.037–8.142; *P* = 0.034) for PFS significantly favoured the use of primary surgery.

In the comparison between surgery alone and surgery plus PORT, the overall survival rates were 92.9% and 91.7%, respectively, with a hazard ratio (HR) of 3.22 (95% CI, 0.27–37.80; *p* = 0.329). Progression-free survival rates were 86.2% and 73.3%, respectively, with a HR of 1.31 (95% CI, 0.35–4.93; *p* = 0.69) (Figure S1).

### Prognostic factors for survival

The value of various clinical prognostic factors in predicting PFS and OS is shown in Table 2. In multivariate analysis, nerve invasion and primary treatment pattern were independent factors associated with PFS, whereas primary treatment pattern was an independent prognostic factor affecting OS (Table 3).

**Table 2:** Univariate analysis results of factors affecting survival

Characteristic	OS (%)			PFS		
	HR	95% CI	*P* value	HR	95% CI	*P* value
Age, y						
≤ 47	1					
> 47	0.894	0.445–1.798	0.754	0.919	0.570–1.484	0.731
Sex						
Male						
Female	0.871	0.430–1.764	0.700	0.800	0.477–1.341	0.397
Alcohol history						
No	1					
Yes	0.485	0.058–4.041	0.504	1.924	0.663–5.579	0.229
Tobacco history						
No	1					
Yes	0.909	0.311–2.660	0.862	0.650	0.340–1.242	0.193
Nerve invasion						
No	1					
Yes	2.208	0.534–9.120	0.274	3.814	1.259–11.557	0.018
Bone invasion						
No	1					
Yes	0.880	0.170–4.545	0.879	0.859	0.264–2.797	0.800
Vessel invasion						
No						
Yes	0.660	0.081–5.411	0.699	2.094	0.656–6.685	0.212
T stage						
T1-T2						
T3-T4	0.771	0.149–3.979	0.756	0.736	0.226–2.399	0.611
Lymph node metastasis						
No						
Yes	0.589	0.285–1.220	0.154	0.886	0.543–1.444	0.627
Primary Surgical treatment						
Yes						
No	5.497	1.007–26.817	0.049	3.042	1.094–8.460	0.033
Concurrent chemotherapy						
No						
Yes	0.838	0.407–1.723	0.630	0.810	0.479–1.367	0.430

**Table 3:** Multivariate analysis results of factors affecting survival

Characteristic	OS (%)			PFS		
	HR	95% CI	*P* value	HR	95% CI	*P* value
Nerve invasion						
No						
Yes			0.274	3.709	1.202–11.447	0.023
Surgical treatment						
Yes						
No	5.497	1.007–26.817	0.049	2.903	1.037–8.142	0.034

### Failure patterns

In total, 19 patients had developed treatment failure by their last follow-up visit. Of the 50 patients, locoregional failure was found in 9 patients. Six of them experienced only local failure, 1 patient developed only neck recurrence, and 2 patients had both. Ten patients failed at distant metastasis, 3 of whom had single organ metastases, and 7 had multiple organ metastases, including 2 with both locoregional recurrence and distant metastasis. The lung was the most common site of metastasis (*n* = 7). Other sites of distant metastasis included the liver (*n* = 4) and bone (*n* = 4).

## Discussion

To our knowledge, the current study is one of the largest single-institution retrospective studies to summarize the clinical features and evaluate the prognostic factors of NACC published to date. Our study indicated that NACC is a rare malignancy with different biological behaviour from common nasopharyngeal cancer. The 5-year overall survival rate (OS) and progression-free survival rate (PFS) were 83.9% and 67.5%, respectively. Primary surgery resulted in a significant overall survival advantage compared with primary RT in patients with NACC. This result also corresponds to the improved progression-free survival in the primary surgery group compared with the primary RT group. Cranial nerve invasion and treatment strategies might be important factors affecting the survival of patients with NACC.

NACC accounts for a small proportion of primary nasopharyngeal malignancies. Given its rarity, the long-term survival outcome of patients with NACC has not been well reported. Management decisions are further complicated due to the lack of consensus regarding the optimal treatment regimen. Adenoid cystic carcinoma arises mainly from secretory glands, most commonly the major and minor salivary glands of the oral and maxillofacial region, whereas it rarely occurs in the nasopharyngeal region. This study showed that NACC accounted for only 0.084% of all malignant neoplasms in the nasopharynx. Additionally, unlike nonkeratinizing squamous cell carcinoma in the nasopharynx, there is no male preponderance for the development of NACC, and the ratio of females to males is 28:22. Nasopharyngeal carcinoma incidence is higher in males than in females, with a ratio of approximately 2.5 in China in 2015 [18].

Unlike other nasopharyngeal malignancies, NACC has a special biological behaviour of perineural invasion, and 21 (42%) of 50 patients had cranial nerve invasions, including optic nerve, oculomotor nerve, and trigeminal nerve invasions. However, NACC had a lower incidence of cranial nerve involvement (26.9%) in Liu’s study [10]. This result may arise with the advent of more modern imaging technology and a lack of sensitivity and specificity from the older techniques. Furthermore, the tumour is inclined to spread along nerves to the orbit or cranial cavity, which may lead to challenges in resection and poor prognosis. NACC has a strong ability of local invasion, such as in the nasal cavity and base of the skull, which would add difficulty to surgical resection. Unlike undifferentiated nonkeratinized carcinoma, the lymph node metastasis rate is relatively low in NACC. Another study showed a cervical metastasis rate of 3.8%-15% [10], while it occurred in 50% of cases in this study (Table 1), which was still lower than nasopharyngeal cancer (64.1%–88.1%) [10, 19]. Epstein‒Barr virus infection has been reported to have a close relationship with the incidence of undifferentiated carcinoma and nonkeratinizing carcinoma, whereas few studies have reported its relationship with NACC. The nonkeratinizing subtype constitutes most cases of epidemical areas (> 95%), and it is predominantly associated with Epstein‒Barr virus (EBV) infection [20, 21]. However, this study showed that EBV DNA levels in plasma were positive (≥ 4000 copy/mL) in a small group of patients, and the positive percentage was 6%.

NACC characteristically exhibits locally aggressive growth with a unique tendency to invade nerves. They could also spread long distances from the primary location along the nerve sheaths. In some cases, skip involvement could also be seen along the perineural space. Moreover, due to the complex anatomical structure of the nasopharynx, total or near-total resection is difficult to achieve. The presence of a positive surgical margin has been associated with decreased survival [22–24]. Previous studies [25–27] reported that ACC, regardless of primary site, was resistant to radiation. In the study by Liu et al. [10], patients with nasopharyngeal adenoid cystic carcinoma undergoing primary surgery had better disease-free survival (DFS) and OS rates than those who received primary radiotherapy. Many studies [28–30] have reported that the first treatment choice for ACC is radical surgery unless the disease is unresectable. A study [10] that enrolled 26 NACC patients at one institution between 1976 and 2003, including 16 patients who underwent traditional 2D-RT, indicated that there was a significant difference in DFS and OS between the surgical treatment group and nonsurgical treatment group. Meanwhile, NACC patients treated by combined surgery and radiotherapy had better survival outcomes. There are some limitations in these studies, and the possible reasons for the results may be that conventional radiotherapy (2D-RT) techniques were used in these previous studies. However, other studies have shown that radiotherapy is associated with a better prognosis in NACC. A retrospective study by Sandeep et al. [14] showed that concurrent chemoradiotherapy could achieve a better prognosis. The number of patients enrolled in these studies was rather small, and those studies were based on data over a long period, including traditional surgical techniques. In our study, 16 patients with ACC received primary RT, the objective response rate reached 81.3% (13/16), and 70.59% (24/34) of patients achieved negative surgical margins. In addition, the 5-year OS and PFS rates of patients with ACC undergoing primary surgery were better than those of patients who underwent primary RT. The results of the current study were inconsistent with those of some previous studies. The possible reasons for the difference may be the development of endoscopic operation and PORT. It has been suggested that postoperative radiotherapy (PORT) may prolong disease-free survival and improve locoregional control in patients undergoing surgery [31]. Consequently, the combination of surgery with PORT has become the mainstream treatment approach for some advanced-stage patients. However, whether PORT can improve long-term survival in patients with NACC remains to be established. In this study, PORT also failed to improve the survival of patients undergoing surgery.

Liu et al. [10] concluded that patients with cranial nerve invasion, advanced stage and surgery showed a significantly worse OS in univariate analysis. Stage and surgical treatment were independent factors affecting OS in multivariate analysis. The possible reasons for the differences may be that the number of cases was relatively small for multivariate analyses. Multivariate analyses in our study showed that nerve invasion and primary treatment were significant factors associated with PFS, and the primary treatment pattern was a significant factor associated with OS. In the study by Huang et al. [32], neural invasion could be seen early and was an unfavourable prognostic factor associated with decreased survival outcome. A similar result was observed in our study. Patients with no nerve invasion had better 5-year DFS rates than those with nerve invasion at diagnosis (81.2% vs. 62.9%, log-rank *P* = 0.006). Based on the above findings, primary surgery could be considered the first treatment choice for patients with ACC.

Despite the inherent limitations of a single-institution retrospective design, the strength of our study is that it represents the largest cohort of patients with NACC treated with RT or surgical treatment in the IMRT era. Our findings must be further validated in a prospective study that needs multi-institutional participation because of its rarity. There is also a critical need to identify molecular markers of response to treatment to further guide the selection of different therapies and perhaps provide targets for novel therapies for patients with NACC.

## Conclusions

Due to the relative insensitivity to radiation**,** primary surgery seems to provide a better chance of disease control and improved survival in NACC, and postoperative radiotherapy should be performed in advanced stages and for residual tumours. cranial nerve invasion and treatment pattern might be important factors affecting the prognosis of patients with NACC.

### Supplementary Information

**Additional file 1: Table S1.** Characteristics and Clinical Data of the Patients Undergoing Primary Surgery.

**Additional file 2: Figure S1.** The overall survival rate (A) and progression-free survival rate (B) in patients with NACC between Surgery Alone and Surgery + PORT. PORT, postoperative radiotherapy; RT, radiotherapy.

## Data Availability

All analyzed data are included in this published article. The original data are available upon reasonable request to the corresponding author.

## Abbreviations

NACC: Nasopharyngeal adenoid cystic carcinoma
IMRT: Intensity-modulated radiation therapy
RT: Radiotherapy
NPC: Nasopharyngeal carcinoma
ENPG: Endoscopic nasopharyngectomy
CMT: Combined modality therapy
OS: Overall survival
PFS: Progression-free survival
DFS: Disease-free survival
2DRT: 2-Dimensional radiotherapy
ORR: Objective response
PORT: Postoperative radiotherapy
