# Supplementary material for: Outcomes of patients in nasopharyngeal adenoid cystic carcinoma in the IMRT era: a single-center experience
Source: BMC Cancer. 2024 May 10;24:576. doi: 10.1186/s12885-024-12159-z (PMC11084105; doi:10.1186/s12885-024-12159-z)
Supplement: Supplementary file 1 — Additional file 1: Table S1. Characteristics and Clinical Data of the Patients Undergoing Primary Surgery. [file 12885_2024_12159_MOESM1_ESM.docx]

| Patient characteristics | Total n (%) | Surgery n (%) | Surgery +PORT n (%) | **P** |
| --- | --- | --- | --- | --- |
| Age, n(%) |  |  |  | 1.000 |
| ≤47 | 17 (50.00) | 8 (53.33) | 9 (47.37) |  |
| ＞47 | 17 (50.00) | 7 (46.67) | 10 (52.63) |  |
| Tobacco history, n(%) |  |  |  | 1.000 |
| 0 | 31 (91.18) | 14 (93.33) | 17 (89.47) |  |
| 1 | 3 (8.82) | 1 (6.67) | 2 (10.53) |  |
| Alcohol history, n(%) |  |  |  | 0.199 |
| 0 | 27 (79.41) | 10 (66.67) | 17 (89.47) |  |
| 1 | 7 (20.59) | 5 (33.33) | 2 (10.53) |  |
| Sex, n(%) |  |  |  | 0.034 |
| Female | 21 (61.76) | 6 (40.00) | 15 (78.95) |  |
| Male | 13 (38.24) | 9 (60.00) | 4 (21.05) |  |
| Nerve Invation, n(%) |  |  |  | 0.296 |
| 0 | 21 (61.76) | 11 (73.33) | 10 (52.63) |  |
| 1 | 13 (38.24) | 4 (26.67) | 9 (47.37) |  |
| Bone Invation, n(%) |  |  |  | 0.030 |
| 0 | 11 (32.35) | 8 (53.33) | 3 (15.79) |  |
| 1 | 23 (67.65) | 7 (46.67) | 16 (84.21) |  |
| T, n(%) |  |  |  | 0.068 |
| T1-T2 | 10 (29.41) | 7 (46.67) | 3 (15.79) |  |
| T3-T4 | 24 (70.59) | 8 (53.33) | 16 (84.21) |  |
| Lymph node metastasis, n(%) |  |  |  | 0.314 |
| 0 | 19 (55.88) | 10 (66.67) | 9 (47.37) |  |
| 1 | 15 (44.12) | 5 (33.33) | 10 (52.63) |  |

**Table S1** Characteristics and Clinical Data of the Patients Undergoing Primary Surgery
