# Supplementary figures and images for: Outcomes of patients in nasopharyngeal adenoid cystic carcinoma in the IMRT era: a single-center experience
Source: BMC Cancer. 2024 May 10;24:576. doi: 10.1186/s12885-024-12159-z (PMC11084105; doi:10.1186/s12885-024-12159-z)

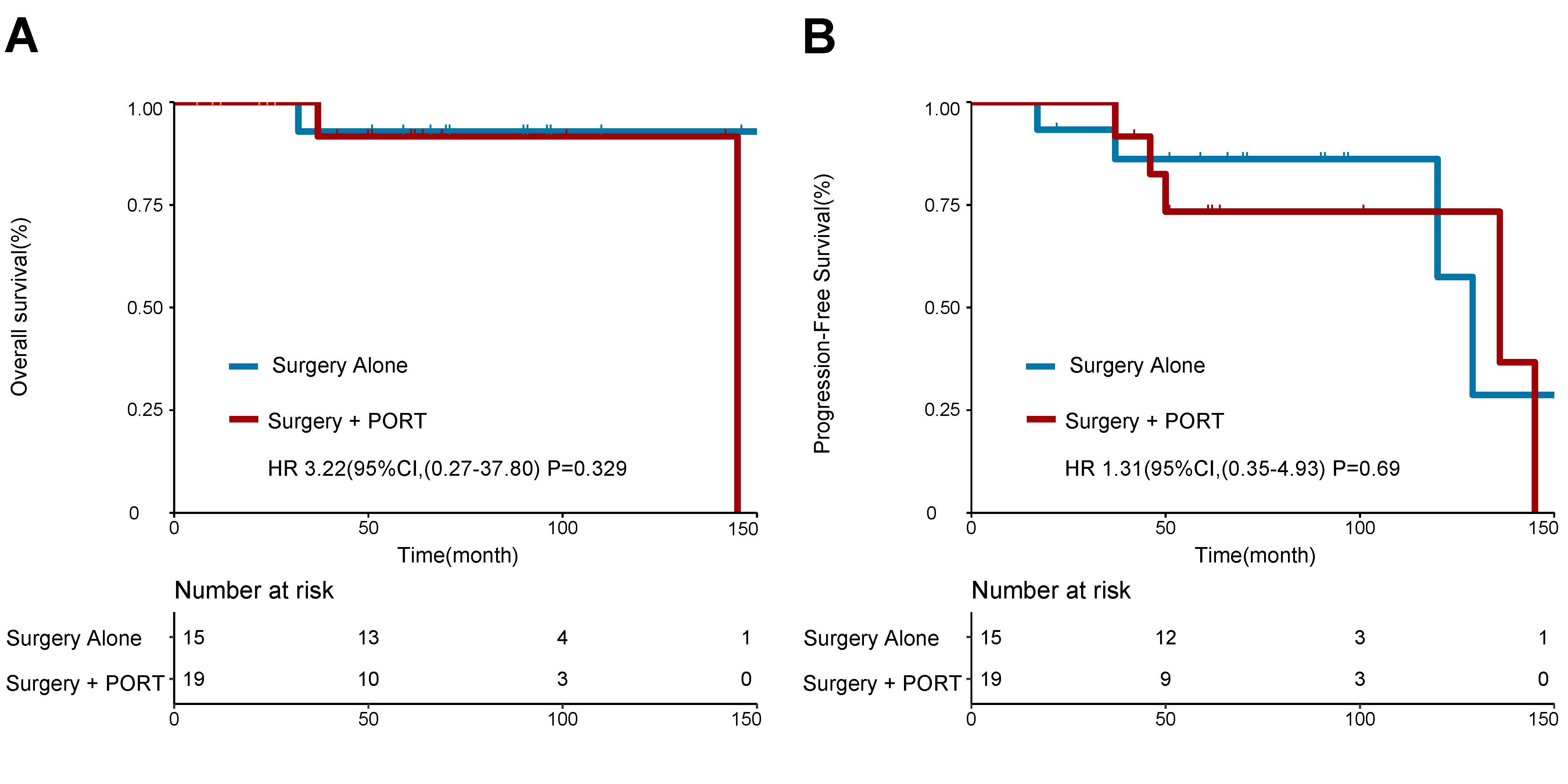

Supplement: Supplementary file 2 — Additional file 2: Figure S1. The overall survival rate (A) and progression-free survival rate (B) in patients with NACC between Surgery Alone and Surgery + PORT. PORT, postoperative radiotherapy; RT, radiotherapy. [file 12885_2024_12159_MOESM2_ESM.jpg]
